# Association between different composite dietary antioxidant indexes and constipation in American male adults: a cross-sectional study

**DOI:** 10.3389/fnut.2024.1404400

**Published:** 2024-07-18

**Authors:** Wei Sun, Yuchao Wang, Lu Han, Yinshi Liu, Hongru Liu, Yunbing Tong, Ziying Jiang, Chen Xu, Daqing Sun

**Affiliations:** ^1^Department of Pediatric Surgery, Tianjin Medical University General Hospital, Tianjin, China; ^2^Faculty of Arts and Science, University of Toronto, Toronto, ON, Canada; ^3^The First Clinical Medical School, Shanxi Medical University, Taiyuan, China; ^4^Department of Colorectal Surgery, Tianjin Union Medical Center, Tianjin, China

**Keywords:** antioxidant, dietary complex antioxidant index, constipation, National Health and Nutrition Examination Survey, oxidative stress

## Abstract

**Background:**

Oxidative stress is acknowledged as a pivotal factor in the intricate pathophysiological processes and pathogenesis of constipation. Modifying dietary patterns can elevate *in vivo* antioxidant biomarker levels, consequently mitigating oxidative stress. The Composite Dietary Antioxidant Index (CDAI) provides a dependable scoring mechanism for quantifying the potential antioxidant capacity of diets. The association between CDAI levels and the risk of constipation remains uncertain.

**Purpose:**

To investigate the potential correlation between CDAI and constipation, aiming to improve constipation management through dietary guidance.

**Methods:**

A total of 11,165 adults aged ≥20 years, drawn from the 2005–2010 National Health and Nutrition Examination Survey, were enrolled in this cross-sectional study. We evaluated the correlation between CDAI levels and the risk of constipation through three weighted logistic regression models. Restricted cubic spline (RCS) analysis was employed to assess nonlinear trends, and stratified analyses were conducted.

**Results:**

After adjusting for all confounding variables, the findings revealed an association between CDAI and constipation [OR = 0.937; 95% CI (0.892, 0.984), *p* = 0.012]. Moreover, individuals in the highest quartile of CDAI demonstrated a 40.1% lower likelihood of experiencing constipation compared to those in the lowest quartile [OR = 0.599; 95% CI (0.382, 0.939), *p* = 0.027]. The RCS analysis indicated a linear relationship between CDAI and constipation (P-non-linear =0.1016). Subgroup analysis by gender revealed a negative correlation in the male population [OR = 0.871; 95% CI (0.801, 0.947), *p* = 0.002], with men in the highest CDAI quartile exhibiting a 59.8% lower likelihood of experiencing constipation compared to those in the lowest quartile [OR = 0.402; 95% CI (0.206, 0.787), *p* = 0.010]. Furthermore, alterations in selenium [OR = 0.997; 95% CI (0.995, 1.000), *p* = 0.039] per milligram were independently linked to constipation. In a gender subgroup analysis of a single antioxidant, changes per milligram of vitamin E [OR = 0.904; 95% CI (0.838 to 0.975), *p* = 0.011] among males were independently associated with constipation.

**Conclusion:**

The fully adjusted model showed a correlation between CDAI and constipation and a significant correlation in quartiles. Meanwhile, subgroup analysis by gender showed that CDAI was negatively associated with constipation in the male population. Moreover, the findings of this study imply that investigations into antioxidant diets should be contextualized within dietary patterns.

## 1 Introduction

Constipation represents a widespread concern afflicting individuals across all age groups (1). It is commonly described as bowel movements that are infrequent or difficult (2). This imposes a significant health burden on patients, significantly impacting their quality of life and mental health (3). Within the community, the median prevalence of constipation among adults is 16%., This prevalence increases significantly among older individuals, reaching 33.5% among adults aged 60 to 101 years (4, 5). Its prevalence is greater among non-Caucasian populations, inpatients, and women; the median prevalence ratio in women to men is 1.5:1 (6). Constipation is a multifactorial disorder with common risk factors such as genetic predisposition, socioeconomic status, parental education, lifestyle, medications, and depression (7, 8). Among them, lifestyle is a more controllable risk factor, especially the relationship between dietary composition and constipation. Recent research indicates that levels of oxidative stress have increased in both constipated patients and animal models (9). Moreover, multiple studies have demonstrated an imbalance of oxidative stress in the intestines of constipated patients (10, 11). Diet plays a crucial role in providing exogenous antioxidants, effectively increasing the levels of endogenous antioxidant biomarkers in the body, thereby alleviating oxidative stress (12, 13). Vitamins A or carotenoids, along with vitamins C and E, as well as other antioxidants, are believed to ameliorate constipation by thwarting cellular damage induced by oxidative stress. Recently, studies have reported that soluble fiber and trace elements, such as selenium, magnesium, and phosphorus, have been thought to reduce the risk of constipation (8, 14–16), while high saturated fat or relatively low energy intake in women are associated with an increased risk (17, 18). Therefore, research by adjusting the level of antioxidants in the diet offers hope for the prevention and control of constipation.

the Composite Dietary Antioxidant Index (CDAI) is a comprehensive score used to assess an individual’s total dietary antioxidant capacity (TAC), which is based on various dietary vitamins and minerals with antioxidant properties, such as vitamins A, C, and E, as well as minerals like selenium and zinc (19, 20). The CDAI serves as a tool to quantify the potential antioxidant capacity of one’s daily diet (21). Existing literature suggests that CDAI is negatively correlated with osteoporosis (22), hypertension (23), depression (20), and cardiovascular disease mortality (24). Furthermore, it is also associated with specific inflammatory biomarkers such as IL-1β and TNFα (25), which are known to be elevated in constipated populations (26). Additionally, there is a noteworthy bimodal distribution observed in constipation and oxidative balance, contingent upon gender disparities (27). Currently, the relationship between CDAI and constipation remains unexplored. Therefore, we conducted a cross-sectional study using data from the National Health and Nutrition Examination Survey (NHANES) from 2005 to 2010. NHANES is a comprehensive representative survey of the U.S. populace, aimed at investigating the possible link between CDAI and the occurrence of constipation, with the objective of enhancing its management through dietary guidance.

## 2 Materials and methods

### 2.1 Data source

NHANES is a major program conducted by the National Center for Health Statistics (NCHS) to collect nationally representative health data concerning the broader population of the United States. NHANES employs a sophisticated multistage stratified probability sampling method, conducting an annual cross-sectional survey on approximately 5,000 individuals. These surveys have been biennially published since 1999. To ensure a representative sample, NHANES deliberately oversamples specific subgroups of the population. Thus, during data analysis, we considered sample weights to correct for differences in the probability of different selections, to compensate for possible underrepresentation of eligible populations, and to adjust for non-coverage and non-response. The NHANES protocol received approval from the NCHS Research Ethics Review Board, and all participants provided informed consent. The NHANES survey data, comprehensive survey operation manuals, consent documents, and brochures for each survey period were made publicly accessible on the NHANES website.^1^

### 2.2 Study participants

**Figure 1:**
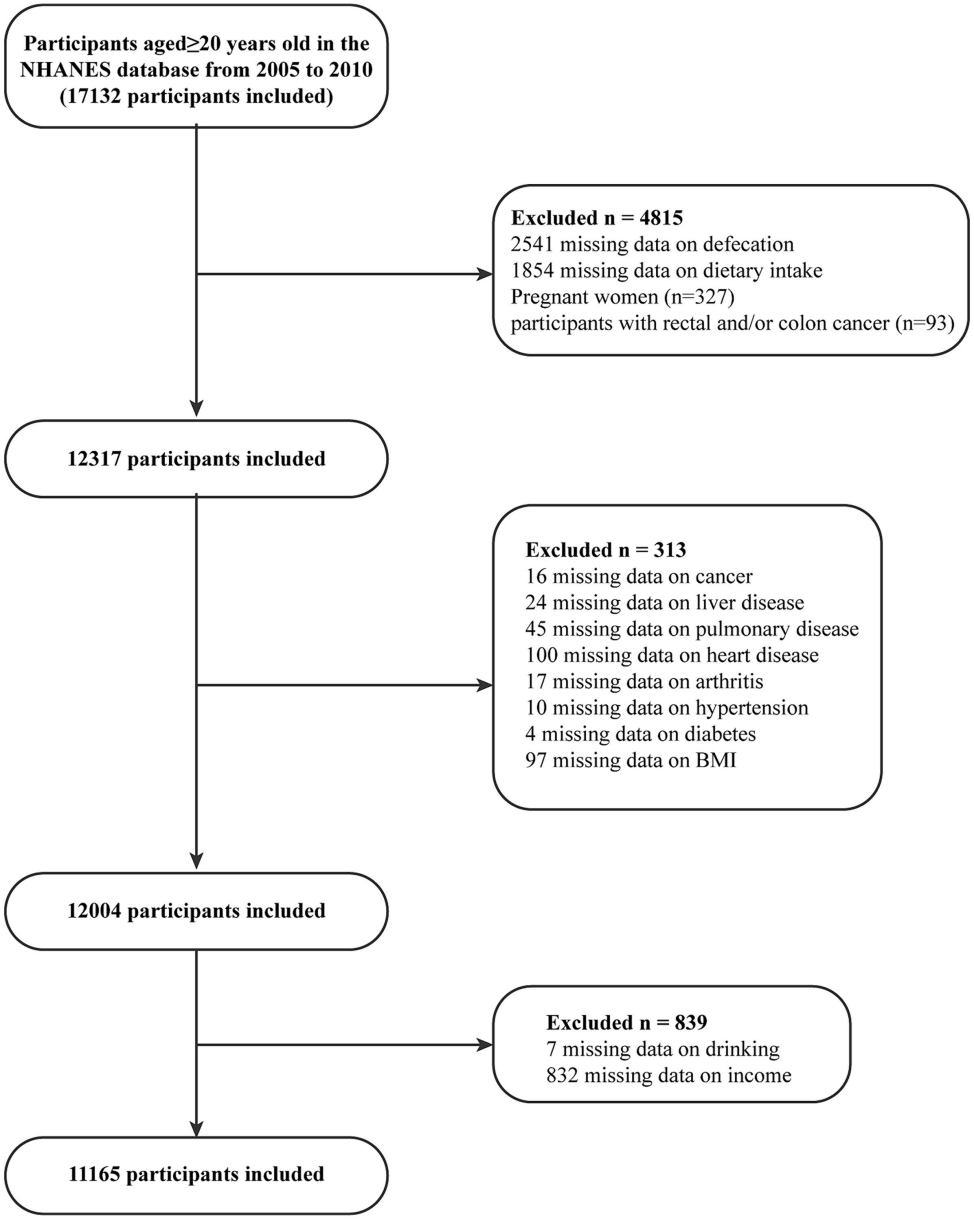
Flowchart depicting participant selection in the study.

In this study, we integrated survey data from the years 2005–2006, 2007–2008, and 2009–2010, as these three cycles included the most comprehensive Bowel Health Questionnaire (BHQ) to date. The data analyzed in this paper were exclusively extracted from adults aged ≥20 years. Participants with colorectal cancer or pregnancy were excluded from the study, as were those with missing covariate data. Ultimately, a total of 11,165 participants were included in the analysis. The process of patient selection is depicted using a flowchart, as illustrated in Figure 1.

### 2.3 Constipation definition

According to previous studies, bowel habits were assessed using the Bristol Stool Form Scale (BSFS), which consists of illustrated and textual descriptions of seven types of stools. Participants were instructed to review the cards and subsequently indicate the number associated with the stool type they typically or most commonly experience to researchers. “Constipation” was defined as type 1 (separate hard lumps, like nuts) or type 2 (sausage-like, but lumpy). As in other NHANES publications, normal stool consistency is defined as types 3, 4, and 5 of the BSFS. To compare the definition of constipation based on stool characteristics with other definitions, we also assessed bowel frequency through the following question from the questionnaire: “How many times do you usually have bowel movements in a week?.” Responses ranged from once a week to 70 times a week. The results of bowel frequency were separated into two categories: those having fewer than three bowel movements per week (constipation), and those with three or more bowel movements per week (non-constipation) (14, 18).

### 2.4 Dietary assessment

NHANES employs non-consecutive 24-h dietary recall to collect participants’ food intake data. The initial dietary recall is conducted at a Mobile Examination Center (MEC), while the second interview takes place via telephone 3 to 10 days later. The daily average antioxidant intake is calculated based on the dietary recall data from these 2 days, and all participants’ CDAI levels are computed using a modified version developed by Wright et al. (14). The CDAI is computed as the sum of the daily average intake of zinc, selenium, carotenoids, vitamins A, C, and E, which is initially standardized by subtracting the mean and then dividing by their standard deviation (SD):


$$\mathrm{CDAI}=\overset{n=6}{\underset{i=1}{\sum }}\left(\mathrm{IndividualIntake}-\mathrm{Mean}\right)/SD$$

### 2.5 Covariates

In order to reduce potential confounding biases in the analysis, we selected covariates based on previous research and clinical rationale. The chosen covariates comprise age, gender, race, education level, family poverty income ratio (PIR), body mass index (BMI), whether they perform vigorous physical activity, smoking status, alcohol consumption, diabetes status, hypertension status, arthritis status, heart disease status, lung disease status, liver disease status, cancer status, dietary fiber intake, total fat and protein intake, carbohydrate intake and water intake. Detailed measurement procedures can be found on https://www.cdc.gov/nchs/nhanes.

### 2.6 Statistical analysis

Due to the complex nationwide sampling design, we only considered 2-year dietary weights (WTDR2D), sampling units (SDMVPSU), and strata (SDMVSTRA) in all analyses. In this instance, new 6-year weights can be calculated by dividing the 2-year weights by 3. A descriptive analysis was performed for all participants. Continuous data were analyzed using means and standard deviations (SD), while categorical variables were expressed as percentages (%). The chi-square test was employed for categorical variables, whereas the t-test was utilized for continuous variables (such as age). The relationship between constipation and CDAI was analyzed using logistic regression models. Three different models, including both unadjusted and multivariable-adjusted models, were employed: Model I (without adjusting for any covariates), Model II (adjusted for gender, age, and race), Model III(adjusted for Model II plus education level, PIR, vigorous physical activity, BMI, diabetes, hypertension, arthritis, heart disease, lung disease, liver disease, cancer, and other covariates). Statistical significance was determined by comparing adjusted odds ratios (ORs) to 1.0 and its 95% confidence intervals (CIs). All statistical analyses were performed using R version 4.2.1(R Foundation for Statistical Computing, Vienna, Austria; http://www.r-project.org), and the statistical significance threshold was ascertained by a two-sided *p*-value of <0.05.

## 3 Results

### 3.1 Demographics

This study analyzed a total of 11,165 eligible participants. Table 1 showcases the baseline characteristics of the study cohort stratified by quartiles of CDAI, unveiling notable disparities across diverse demographic and lifestyle factors including gender, race, education level, family poverty income ratio (PIR), vigorous physical activity, smoking status, alcohol consumption, diabetes, pulmonary disease, dietary fiber intake, total fat intake, protein intake, carbohydrate intake, and moisture intake (*p* < 0.001). Particularly noteworthy is the observation that individuals in the uppermost quartile of CDAI were predominantly between 40 to 60 years of age male, of non-Hispanic White ethnicity, possessed higher levels of education, exhibited greater economic prosperity, maintained a BMI within the range of 25 to 30, participated in vigorous physical activity, refrained from smoking and consuming alcohol. Additionally, a notable correlation was identified between CDAI quartiles and constipation even in the absence of covariate adjustment (*p* < 0.001).

**Table 1:** Demographic characteristics stratified by Quartile of CDAI (*n* = 11,165).

Characteristic	*N*^1^	Overall, *N* = 11,165 (100%)^2^	Q1, *N* = 2,792 (22%)^2^	Q2, *N* = 2,791 (24%)^2^	Q3, *N* = 2,791 (27%)^2^	Q4, *N* = 2,791 (28%)^2^	*P* value^3^
Age (years)	11,165						**0.012**
20–40		3,615 (37.08%)	913 (40.33%)	858 (35.72%)	898 (34.91%)	946 (37.80%)	
40–60		3,789 (39.40%)	872 (35.15%)	946 (38.85%)	971 (41.44%)	1,000 (41.24%)	
> = 60		3,761 (23.52%)	1,007 (24.53%)	987 (25.43%)	922 (23.64%)	845 (20.96%)	
Sex	11,165						**<0.001**
Female		5,664 (51.79%)	1,852 (70.52%)	1,563 (58.67%)	1,299 (47.97%)	950 (34.78%)	
Male		5,501 (48.21%)	940 (29.48%)	1,228 (41.33%)	1,492 (52.03%)	1,841 (65.22%)	
Race	11,165						**<0.001**
Mexican American		1,884 (7.56%)	517 (8.15%)	509 (8.69%)	433 (7.03%)	425 (6.64%)	
Other Hispanic		865 (3.99%)	276 (5.24%)	213 (4.14%)	185 (3.22%)	191 (3.61%)	
Non-Hispanic White		5,793 (72.64%)	1,256 (67.12%)	1,409 (70.78%)	1,550 (74.99%)	1,578 (76.34%)	
Non-Hispanic Black		2,201 (10.75%)	648 (14.15%)	560 (11.65%)	505 (9.75%)	488 (8.26%)	
Other Race - Including Multi-Racial		422 (5.06%)	95 (5.34%)	100 (4.74%)	118 (5.02%)	109 (5.15%)	
Education level	11,165						**<0.001**
<=High school		5,557 (40.95%)	1,742 (55.15%)	1,455 (43.93%)	1,238 (36.09%)	1,122 (31.87%)	
>High school		5,608 (59.05%)	1,050 (44.85%)	1,336 (56.07%)	1,553 (63.91%)	1,669 (68.13%)	
Family PIR	11,165						**<0.001**
<2		4,958 (32.16%)	1,595 (44.14%)	1,269 (34.02%)	1,068 (27.09%)	1,026 (25.98%)	
> = 2		6,207 (67.84%)	1,197 (55.86%)	1,522 (65.98%)	1,723 (72.91%)	1,765 (74.02%)	
BMI	11,165						**0.004**
Under/normal weight		3,156 (31.21%)	775 (33.21%)	752 (28.50%)	785 (29.52%)	844 (33.61%)	
Overweight		3,823 (33.51%)	936 (32.39%)	922 (31.97%)	980 (34.80%)	985 (34.47%)	
Obese		4,186 (35.28%)	1,081 (34.40%)	1,117 (39.53%)	1,026 (35.68%)	962 (31.92%)	
Vigorous physical activity	11,165	3,750 (38.95%)	718 (29.51%)	813 (33.48%)	989 (41.20%)	1,230 (48.93%)	**<0.001**
Smoking status	11,164						**<0.001**
Never		5,869 (53.25%)	1,349 (47.50%)	1,478 (53.10%)	1,535 (56.04%)	1,507 (55.23%)	
Former		2,903 (24.97%)	636 (20.66%)	723 (24.48%)	757 (25.61%)	787 (28.17%)	
Current		2,392 (21.78%)	806 (31.84%)	590 (22.43%)	499 (18.36%)	497 (16.60%)	
Alcohol intake	11,165	9,747 (89.57%)	2,300 (85.28%)	2,433 (89.32%)	2,496 (90.82%)	2,518 (91.97%)	**<0.001**
Diabetes	11,165	1,276 (7.71%)	368 (8.18%)	355 (9.59%)	303 (7.80%)	250 (5.64%)	**<0.001**
Hypertension	11,165	3,942 (29.94%)	1,049 (30.58%)	1,014 (31.74%)	976 (30.06%)	903 (27.77%)	0.10
Arthritis	11,165	3,158 (24.66%)	893 (28.24%)	786 (24.40%)	774 (24.10%)	705 (22.60%)	**0.013**
Heart disease	11,165	921 (6.02%)	278 (6.70%)	246 (7.26%)	207 (5.61%)	190 (4.81%)	**0.016**
Pulmonary disease	11,165	1,960 (17.58%)	557 (21.77%)	494 (18.53%)	483 (17.00%)	426 (14.02%)	**<0.001**
Liver disease	11,165	379 (3.09%)	85 (2.74%)	100 (3.26%)	101 (3.32%)	93 (3.02%)	0.8
Cancer	11,165	1,048 (8.57%)	245 (8.65%)	253 (8.05%)	276 (9.10%)	274 (8.44%)	0.8
Dietary fiber intake	11,165	15.05 (10.60, 20.75)	9.00 (6.60, 12.05)	12.80 (10.10, 16.30)	16.30 (13.05, 20.80)	21.90 (17.25, 27.70)	**<0.001**
Total fat intake	11,165	73.23 (53.04, 99.54)	49.94 (36.61, 64.07)	67.54 (52.46, 85.09)	80.66 (61.69, 102.62)	102.19 (74.25, 133.24)	**<0.001**
Protein intake	11,165	77.26 (58.35, 101.10)	51.76 (41.55, 62.62)	69.72 (57.69, 83.33)	85.11 (70.51, 101.15)	109.14 (88.06, 136.86)	**<0.001**
Carbohydrate intake	11,165	236.58 (181.28, 308.40)	172.90 (132.93, 217.49)	214.38 (174.43, 266.76)	252.95 (204.86, 310.24)	310.45 (246.75, 400.48)	**<0.001**
Moisture intake	11,165	2,715.62 (2,058.40, 3,595.27)	2,093.18 (1,590.48, 2,810.95)	2,462.05 (1,913.82, 3,245.37)	2,806.84 (2,216.41, 3,560.32)	3,378.96 (2,673.46, 4,266.08)	**<0.001**
Constipation	11,165						**<0.001**
CON		10,049 (90.28%)	2,397 (83.46%)	2,484 (89.45%)	2,538 (91.72%)	2,630 (95.00%)	
STC		1,116 (9.72%)	395 (16.54%)	307 (10.55%)	253 (8.28%)	161 (5.00%)	

^1^*N* not missing (unweighted). ^2^Mean (SD) for continuous; *n* (%) for categorical. ^3^Chi-squared test with Rao and Scott’s second-order correction; Wilcoxon rank-sum test for complex survey samples.Statistically significant *p*-values are in bold.

### 3.2 The relationship between CDAI levels and constipation

**Figure 2:**
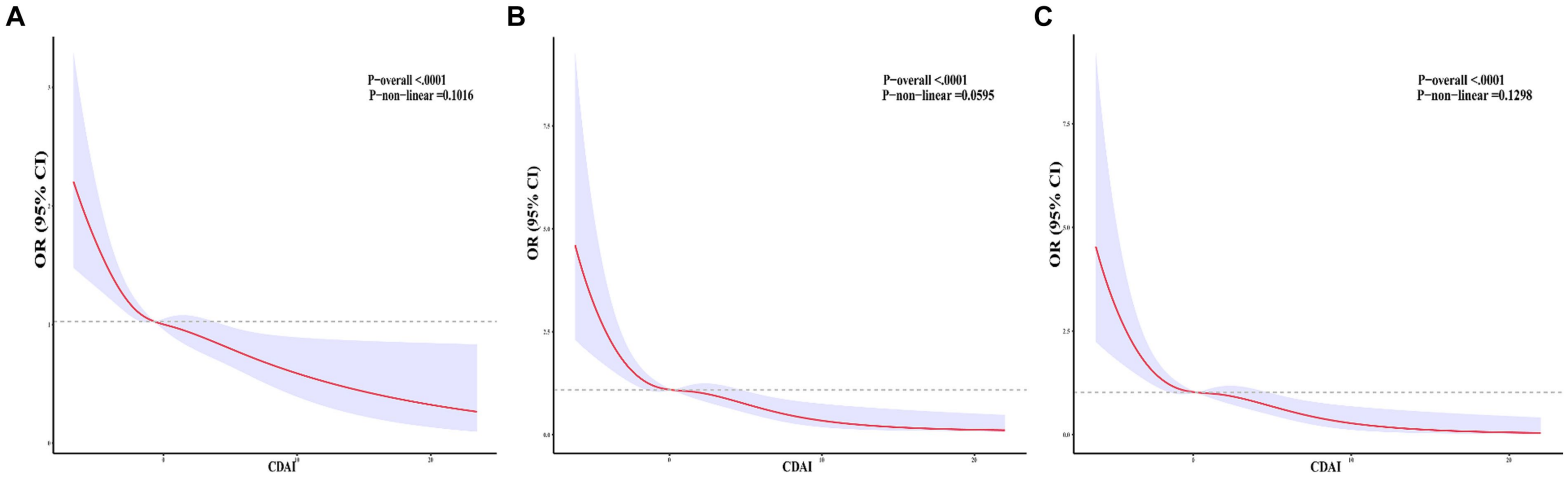
Correlations between CDAI and constipation. **(A)** A linear correlation between CDAI and constipation. **(B)** A linear relationship between CDAI and male patients with constipation. **(C)** A linear relationship between CDAI and female patients with constipation.

Table 2 illustrates the weighted logistic regression models depicting the relationship between CDAI and constipation. In models I, II, and III, there is a negative association between CDAI and constipation [OR = 0.858; 95% CI (0.830, 0.886); *p* < 0.001; OR = 0.894; 95% CI (0.864, 0.925); p < 0.001; OR = 0.937; 95% CI (0.892, 0.984); *p* = 0.012], suggesting that each unit increase in CDAI is associated with a decreased likelihood of constipation. Following the categorization of CDAI into quartiles, individuals in the highest quartile exhibited a 40.1% reduced likelihood of experiencing constipation in contrast to those in the lowest quartile [OR = 0.599; 95% CI (0.382, 0.939); *p* = 0.027]. The restricted cubic splines (RCS) of Figure 2 reveal a linear correlation between CDAI and constipation (non-linear *p* = 0.1016). Table 2 displays the results of stratified analysis conducted according to gender differences. In male patients, CDAI demonstrates a negative association with constipation [OR = 0.871; 95% CI (0.801, 0.947); *p* = 0.002]. Moreover, males in the highest quartile of CDAI exhibit a 59.8% reduced likelihood of experiencing constipation compared to those in the lowest quartile [OR = 0.402; 95% CI (0.206, 0.787); *p* = 0.010]. In models I and II, a significant association between CDAI and constipation in female patients was observed (*p* < 0.001); however, after adjusting for all confounding factors, no correlation was found between CDAI and constipation in female patients (*p* > 0.05).

**Table 2:** The association between composite dietary antioxidant index and constipation.

	Model I	Model II	Model III
	OR (95%CI) *p*-value	OR (95%CI) *p*-value	OR (95%CI) *p*-value
Composite dietary antioxidant index			
CDAI (continuity value)	0.858 (0.830, 0.886) **<0.001**	0.894 (0.864, 0.925) **<0.001**	0.937 (0.892, 0.984) **0.012**
Quartile 1	Ref	Ref	Ref
Quartile 2	0.595 (0.467, 0.759) **<0.001**	0.667 (0.517, 0.859) **<0.001**	0.810 (0.593, 1.106) 0.174
Quartile 3	0.455 (0.356, 0.582) **<0.001**	0.569 (0.434, 0.746) **<0.001**	0.792 (0.542, 1.158) 0.215
Quartile 4	0.2653 (0.206, 0.342) **<0.001**	0.376 (0.282, 0.501) **<0.001**	0.599 (0.382, 0.939) **0.027**
*P* for trend	**<0.001**	**<0.001**	**0.036**
**Stratified by sex**			
Male			
CDAI (Continuity Value)	0.841 (0.788, 0.898) **<0.001**	0.849 (0.796, 0.906) **<0.001**	0.871 (0.801, 0.947) **0.002**
Quartile 1	Ref	Ref	Ref
Quartile 2	0.568 (0.361, 0.895) **0.016**	0.588 (0.371, 0.932) **0.025**	0.588 (0.371, 0.932) 0.058
Quartile 3	0.382 (0.225, 0.649) **<0.001**	0.412 (0.235, 0.720) **0.003**	0.412 (0.235, 0.720) **0.041**
Quartile 4	0.230 (0.135, 0.393) **<0.001**	0.248 (0.144, 0.428) **<0.001**	0.248 (0.144, 0.428) **0.01**
*P* for trend	**<0.001**	**<0.001**	**0.013**
Female			
CDAI (continuity value)	0.909 (0.869, 0.951) **<0.001**	0.914 (0.874, 0.956) **<0.001**	0.960 (0.907, 1.016) 0.147
Quartile 1	Ref	Ref	Ref
Quartile 2	0.666 (0.512, 0.865) **0.003**	0.678 (0.519, 0.887) **0.006**	0.819 (0.567, 1.184) 0.273
Quartile 3	0.609 (0.459, 0.809) **0.001**	0.629 (0.471, 0.841) **0.003**	0.851 (0.543, 1.335) 0.466
Quartile 4	0.438 (0.304, 0.632) **<0.001**	0.456 (0.315, 0.662) **<0.001**	0.676 (0.395, 1.158) 0.146
*P* for trend	**<0.001**	**<0.001**	0.183

*p* < 0.05 was set as the threshold of statistical significance and marked in bold values.

### 3.3 The relationship between antioxidant components and constipation

**Figure 3:**
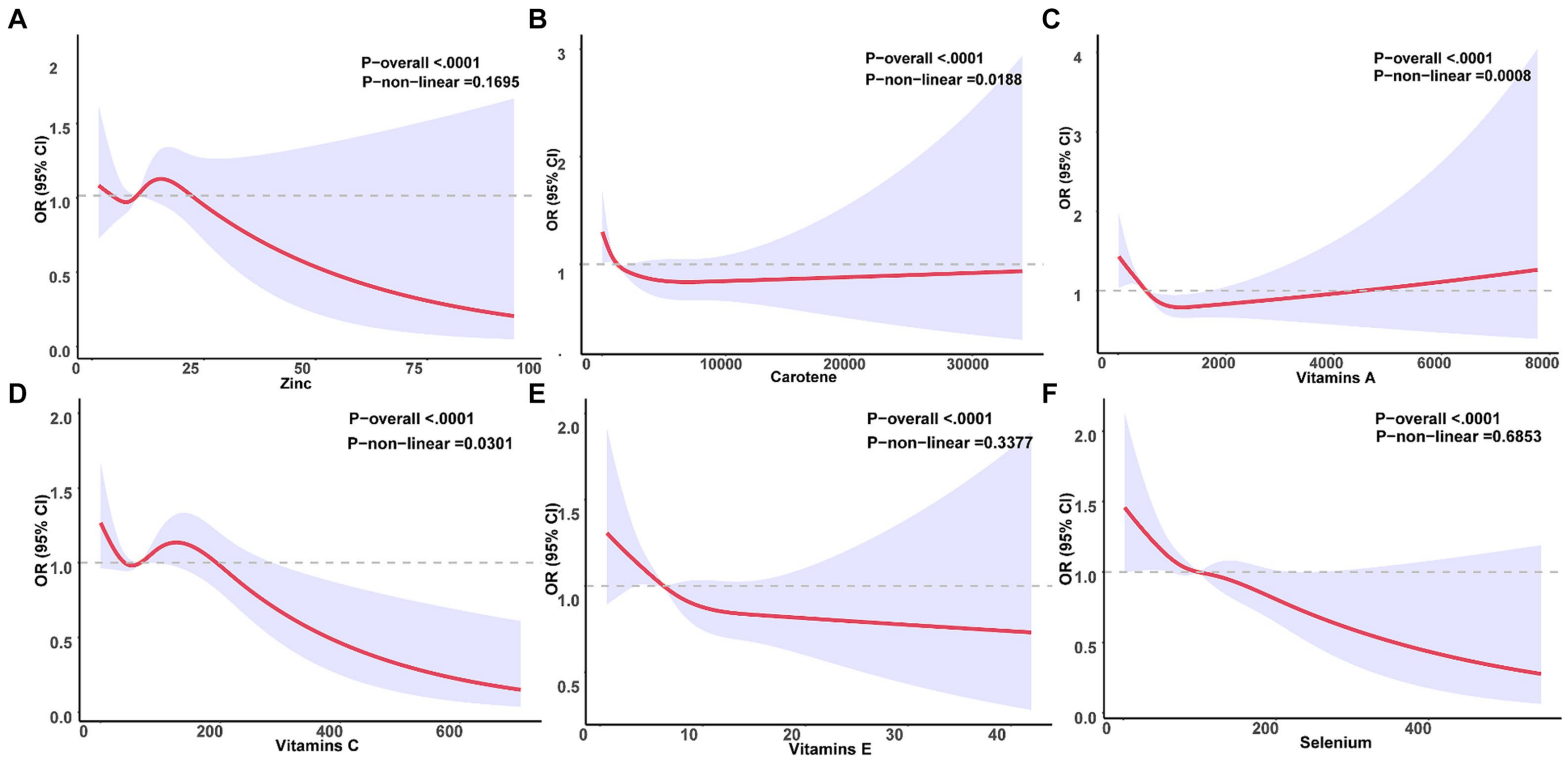
Relationship between six antioxidant components and constipation. A nonlinear correlation was observed between Carotene **(B)**, Vitamin A **(C)**, and Vitamin C **(D)** and the incidence of constipation. Conversely, a linear relationship was identified between Zinc **(A)**, Vitamin E **(E)**, and Selenium **(F)** and constipation.

**Figure 4:**
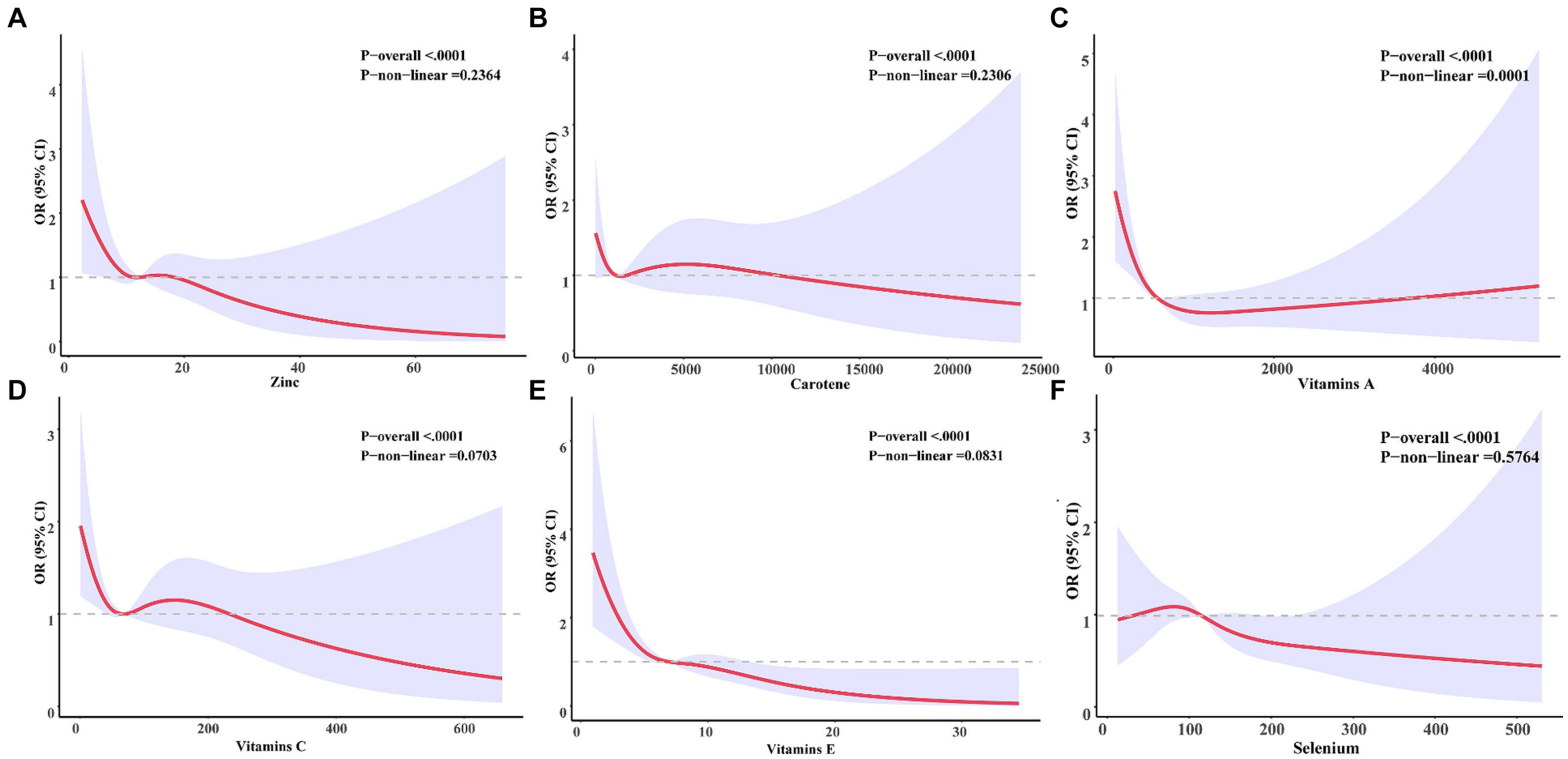
Six antioxidants were associated with a dose–response relationship to constipation in male participants. Zinc **(A)**, Carotene **(B)**, Vitamin A **(C)**, Vitamins C **(D)**, Vitamins D **(E)**, Selenium **(F)**.

**Figure 5:**
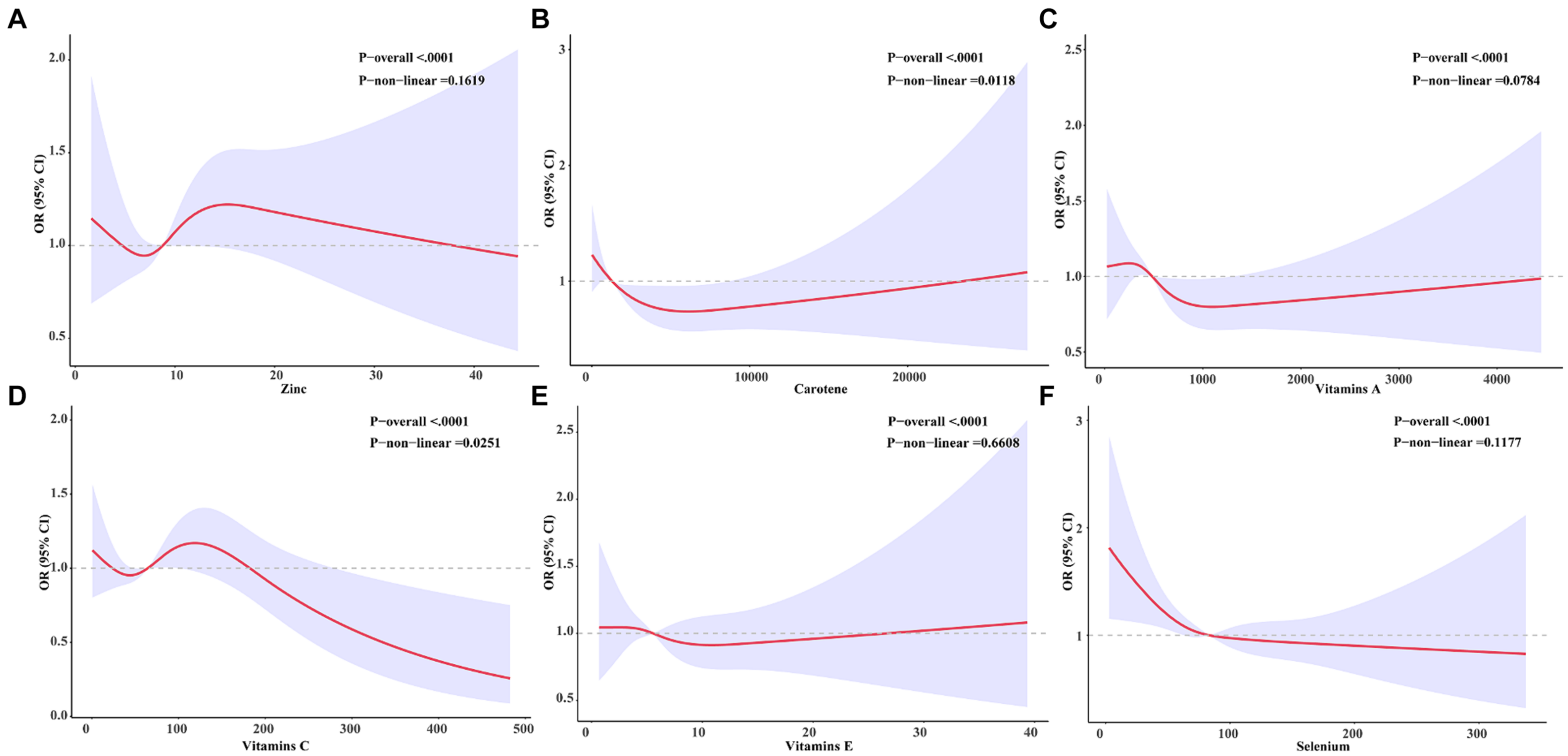
Six antioxidants were associated with a dose–response relationship to constipation in female participants. Zinc **(A)**, Carotene **(B)**, Vitamin A **(C)**, Vitamins C **(D)**, Vitamins D **(E)**, Selenium **(F)**.

We conducted further analysis to examine the relationship between six antioxidant components of CDAI and constipation. As depicted in Table 3, all six antioxidant components were observed to be negatively associated with constipation in models I and II. Nonetheless, following adjustment for all variables, selenium exhibited an independent association with constipation, with an odds ratio of 0.997 [95% CI (0.995, 1.000); *p* = 0.039] per milligram increase. Among men, change per milligram of vitamin E (OR = 0.9039; [95% CI (0.995, 1.000); *p* = 0.039]) was independently associated with constipation risk after adjusting for all variables; however, this association was not observed in women. To delve deeper into the nonlinear dose–response relationship between antioxidant components and constipation, we constructed restricted cubic splines (RCS) for six antioxidant components (vitamins A, C, E, carotenoids, zinc, and selenium) and their association with constipation in Figure 3. The analysis using RCS revealed nonlinear dose–response relationships between carotenoids (non-linear *p* = 0.019), vitamin A (non-linear *p* = 0.001), and vitamin C (non-linear *p* = 0.030) levels and the prevalence of constipation. Simultaneously, we conducted a gender-stratified analysis to elucidate the dose–response relationship between the six antioxidants and constipation among participants of varying genders (Figures 4, 5).

**Table 3:** The association between composite dietary antioxidant index (components) and constipation.

	Model I	Model II	Model III
	OR (95%CI) *p*-value	OR (95%CI) *p*-value	OR (95%CI) P-value
Composite dietary antioxidant index (components)			
+Vitamin A	0.9991 (0.9988, 0.9995) **<0.001**	0.9993 (0.9990, 0.9997) **0.002**	0.9998 (0.9995, 1.0001) 0.213
Vitamin C	0.9970 (0.9958, 0.9982) **<0.001**	0.9974 (0.9903, 0.9986) **<0.001**	0.9989 (0.9976, 1.0003) 0.1363
Vitamin E	0.9025 (0.8775, 0.9283) **<0.001**	0.9292 (0.9042, 0.9548) **<0.001**	0.9791 (0.9491, 1.0101) 0.1756
Zinc	0.9308 (0.9118, 0.9501) **<0.001**	0.9608 (0.9398, 0.9817) **<0.001**	0.9940 (0.9755, 1.0129) 0.521
Selenium	0.9925 (0.9909, 0.9940) **<0.001**	0.9951 (0.9933, 0.9969) **<0.001**	0.9974 (0.9951, 0.9998) **0.0387**
Carotenoid	0.9999 (0.9998, 0.9999) **<0.001**	0.9999 (0.9998, 0.9999) **0.0014**	0.9999 (0.9999, 1.0000) 0.1754
**Stratified by sex**			
**Male**			
Vitamin A	0.9987 (0.9979, 0.9996) **0.005**	0.9989 (0.9981, 0.9998) **0.013**	0.9995 (0.9986,1.0003) 0.1969
Vitamin C	0.9971 (0.9950, 0.9992) **0.009**	0.9967 (0.9946, 0.9988) **0.003**	0.9982 (0.9961,1.0004) 0.1070
Vitamin E	0.8854 (0.8028, 0.9114) **<0.001**	0.8665 (0.8126, 0.9238) **<0.001**	0.9039 (0.8382,0.9748) **0.0109**
Zinc	0.9296 (0.8913, 0.9696) **0.001**	0.9324 (0.8948, 0.9717) **0.012**	0.9708(0.9146,1.0305) 0.316
Selenium	0.9942 (0.9915, 0.9969) **<0.001**	0.9941 (0.9912, 0.9970) **<0.001**	0.9974 (0.9930,1.0018) 0.2397
Carotene	0.9999 (0.998, 0.9999) **0.027**	0.9999 (0.9998, 0.9999) **0.0351**	0.9999 (0.9999,1.0000) 0.4824
**Female**			
Vitamin A	0.9994 (0.9990,0.9999) **<0.001**	0.9995 (0.9991,1.0000) 0.0545	0.9999 (0.9996,1.0000) 0.4954
Vitamin C	0.9977 (0.9962,0.9993) **0.0066**	0.9978 (0.9963,0.9993) **0.0057**	0.9992 (0.9976,1.0009) 0.3975
Vitamin E	0.9497 (0.9202,0.9801) **0.0019**	0.9528 (0.9236,0.9828) **0.0031**	0.9966 (0.9647,0.0283) 0.7992
Zinc	0.9763 (0.9514,1.0020) **<0.001**	0.9773 (0.9519,1.0034) 0.0867	1.0033 (0.9832,1.0238) 0.7349
Selenium	0.9963 (0.9940,0.9986) **0.0022**	0.9959 (0.9935,0.9983) **0.0013**	0.9974 (0.9941,1.0007) 0.1219
Carotene	0.9998 (0.9998,0.9999) **0.0049**	0.9999 (0.9998,0.9999) **0.0105**	0.9999 (0.9999,1.0000) 0.2081

*P* < 0.05 was set as the threshold of statistical significance and marked in bold values.

### 3.4 Subgroup analysis

**Figure 6:**
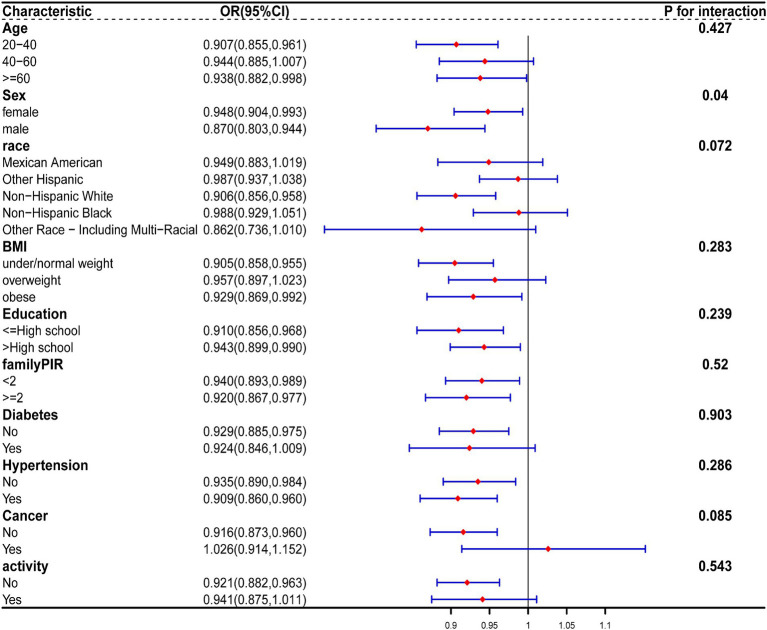
The relationship between CDAI and covariates was assessed by weighted stratified and interaction analysis. The covariates include age, race, BMI, educational levels, smoking, PIR, diabetes, hypertension, cancer, and vigorous physical activity.

**Figure 7:**
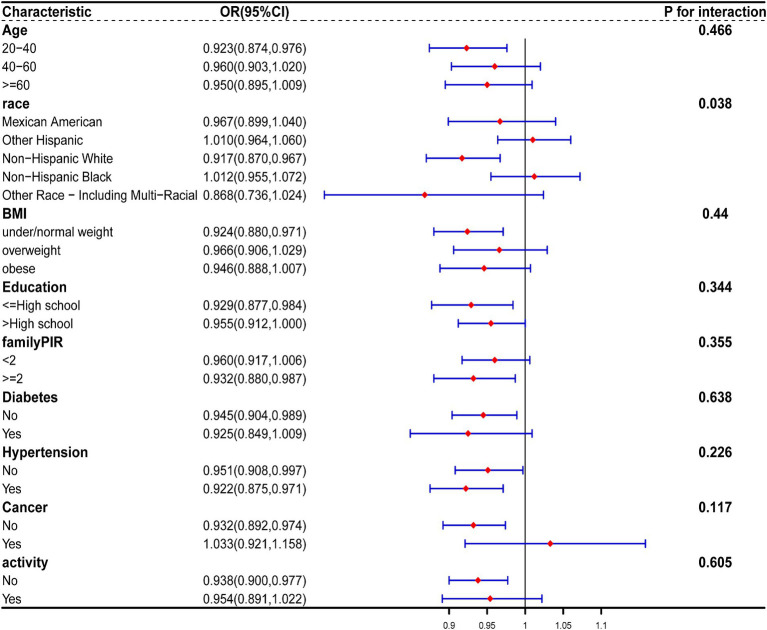
The relationship between CDAI and covariates in men was assessed by weighted stratified and interaction analysis. The covariates include age, race, BMI, educational levels, smoking, PIR, diabetes, hypertension, cancer, and vigorous physical activity.

**Figure 8:**
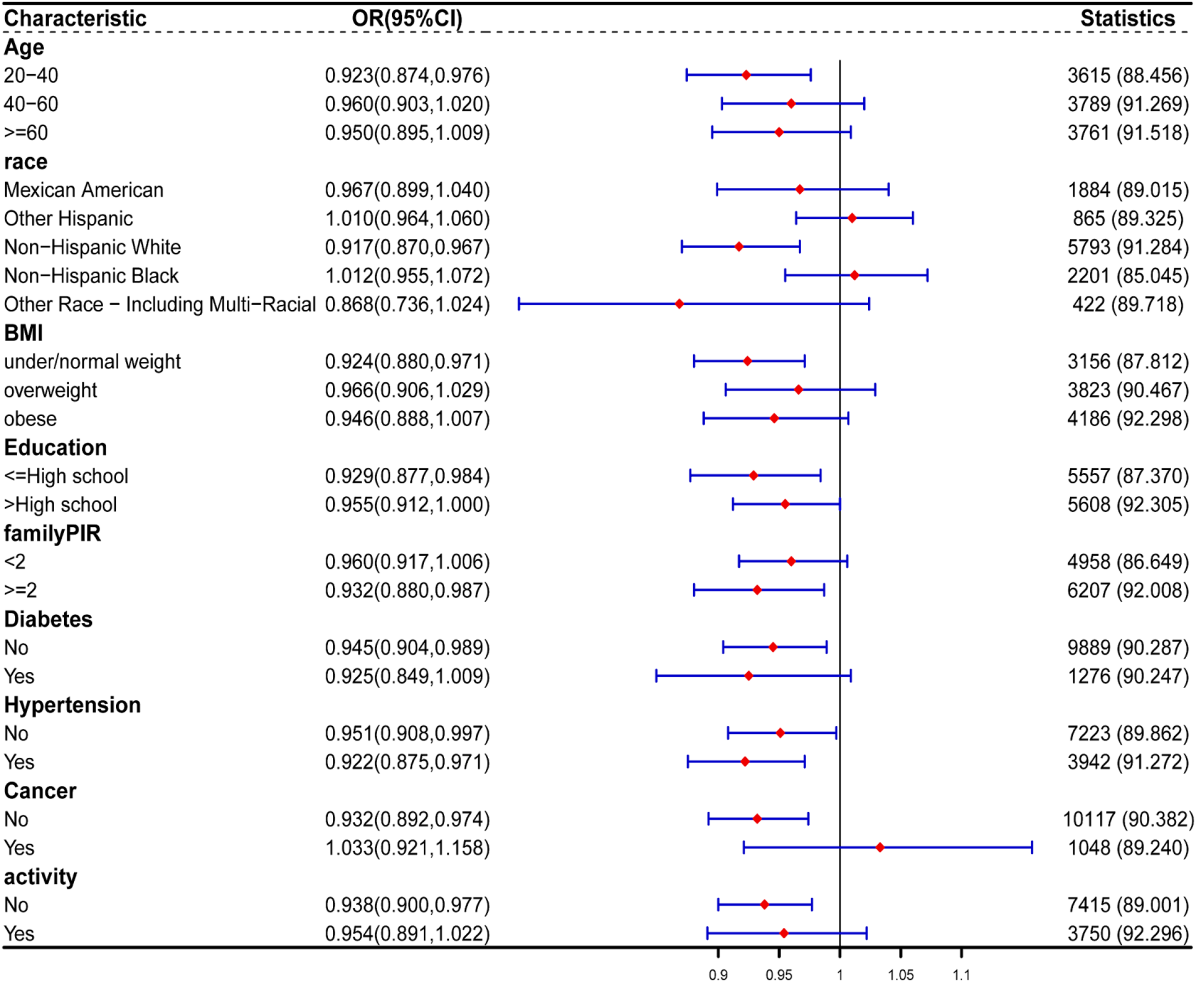
The relationship between CDAI and covariates in women was assessed by weighted stratified and interaction analysis. The covariates include age, race, BMI, educational levels, smoking, PIR, diabetes, hypertension, cancer, and vigorous physical activity.

Stratified analysis was performed based on age, gender, race, BMI, education, family PIR, diabetes, cancer, hypertension, and activity. Forest plots of Figure 6 were generated, which demonstrates a persistent negative correlation between CDAI and the risk of constipation. Simultaneously, we noted that among men, the inverse association between CDAI and constipation risk was notably significant among younger participants and those of non-Hispanic White ethnicity. A similar trend was observed among women. No significant interaction was detected between these variables (all p for interaction >0.05; Figures 7, 8).

## 4 Discussion

Our study’s fully adjusted model III revealed a negative association between CDAI and constipation. Additionally, individuals in the highest quartile of CDAI exhibited a 40.1% reduced likelihood of constipation in comparison to those in the lowest quartile. Nevertheless, subgroup analysis by gender uncovered a negative correlation between CDAI and constipation among males. Males in the highest quartile of CDAI demonstrated a 60% reduced likelihood of constipation compared to their counterparts in the lowest quartile. However, female patients exhibited no association with constipation in the fully adjusted model. Following adjustment for all confounding factors, selenium emerged as a potential independent component associated with constipation. Dose–response analysis indicated a linear relationship between CDAI and constipation.

To the best of our knowledge, our study represents the first cross-sectional investigation exploring the correlation between CDAI levels and constipation. Based on the above, we launched dietary and lifestyle management serves as a primary strategy for preventing and treating constipation. We suggest additional research in this area.

Oxidative stress denotes an imbalance between antioxidants and prooxidants, leading to subsequent tissue and organ damage. The accumulation of reactive oxygen species (ROS) can result in the oxidation of DNA, proteins, carbohydrates, and lipids, which leads to cellular apoptosis and organ dysfunction. However, increasing evidence supports oxidative stress as an important precipitating factor for constipation. Inhibiting oxidative stress can maintain redox homeostasis, thereby alleviating constipation (13, 28). The dietary antioxidant capacity bears significant potential in predicting adult health outcomes (29). Previous studies have demonstrated that crude polysaccharides from *Cistanche deserticola* may alleviate constipation by attenuating oxidative stress to safeguard intestinal neurons (13). Findings from NHANES also indicate an association between low dietary fiber and fluid intake and constipation (30). Several individual components exhibiting antioxidant activity have been investigated for their potential role in constipation in preclinical experiments, including kale (31), a green kiwifruit powder (32), and dragon fruit oligosaccharide (33). Clinical studies have found that prune juice containing sorbitol, pectin, and polyphenol may alleviate constipation (34). The dietary quality has been demonstrated to exhibit a significant relationship with constipation (35). Thus, our study may further bolster the link between antioxidant intake and diet-related constipation. Gender differences also play an important role in the balance of antioxidants (36). Despite the mounting evidence implicating antioxidants in constipation, the influence of gender on the outcomes remains uncertain. A recent study has shown that increasing intake of lycopene can improve intestinal function in men (37), providing further support for the existence of gender differences, as demonstrated by our research. Elevating CDAI levels is inclined to afford protection against constipation symptoms in males but not in females, mirroring the higher prevalence of constipation among females. Furthermore, our further analysis of antioxidant components shows a negative correlation between selenium and constipation. Nutrients and components are consumed in combination and may interact with each other in complex ways (28). The association between individual components and diseases may be difficult to explore and explain (12). Moreover, previous research has mainly focused on individual antioxidants, but the current trend is increasingly recognizing the importance of diet as a whole (13). Consequently, prudence should be exercised when interpreting the effects of individual antioxidant components on diseases.

A notable strength of this study lies in its utilization of NHANES data, acquired through a stratified multistage probability sampling approach, thereby augmenting the reliability and representativeness of the investigation. Furthermore, we accounted for potential confounders, such as gender, age, race, education level, family PIR, BMI, activity level, smoking, and drinking, in our analyses to minimize the effects of confounding variables. However, this study does have some limitations. Firstly, recall-based questionnaire assessment may introduce measurement errors and inaccuracies when assessing antioxidant components. Secondly, bias is inevitable in cross-sectional studies. Moreover, despite adjusting for certain potential confounders, we cannot completely disregard the potential influence of other confounding variables.

## 5 Conclusion

In conclusion, we observed that CDAI acts as a protective factor against constipation in males. Patients with high levels of CDAI tend to have a lower incidence of constipation. There is a linear relationship between the CDAI level and the constipation risk. In addition, we also found that changes per milligram of selenium were independently associated with constipation, and changes per milligram of vitamin E in men were independently associated with constipation. Therefore, We suggest further exploration of antioxidants within dietary patterns and advise careful interpretation of the effects of individual antioxidant components. Furthermore, given the potential of diet as a modifiable intervention with significant health implications, continued exploration in this area is crucial, especially through larger-scale prospective cohort studies.

## Data availability statement

Publicly available datasets were analyzed in this study. This data can be found at: https://www.cdc.gov/nchs/nhanes/index.htm.

## Ethics statement

The studies involving humans were approved by NCHS IRB/ERB Protocol Number: NHANES 2011–2012 (Protocol #2011–17); NHANES 2013–2014 (Continuation of Protocol #2011–17). The patients/participants provided their written informed consent to participate in this study. The studies were conducted in accordance with the local legislation and institutional requirements. Written informed consent for participation was not required from the participants or the participants’ legal guardians/next of kin in accordance with the national legislation and institutional requirements.

## Author contributions

WS: Formal analysis, Methodology, Writing – original draft, Conceptualization, Writing – review & editing, Data curation, Validation, Visualization. YW: Methodology, Writing – review & editing, Conceptualization, Data curation. LH: Writing – review & editing, Visualization. YL: Writing – review & editing, Methodology. HL: Writing – review & editing, Formal analysis. YT: Writing – review & editing, Formal analysis. ZJ: Writing – review & editing, Formal analysis. DS: Funding acquisition, Project administration, Resources, Writing – review & editing, Supervision. CX: Project administration, Writing – review & editing, Supervision.

## Footnotes

^1^ https://www.cdc.gov/nchs/nhanes
