# Corrigendum: Association between different composite dietary antioxidant indexes and constipation in American male adults: a cross-sectional study

**DOI:** 10.3389/fnut.2024.1530119

**Published:** 2024-12-04

**Authors:** Wei Sun, Yuchao Wang, Lu Han, Yinshi Liu, Hongru Liu, Yunbing Tong, Ziying Jiang, Chen Xu, Daqing Sun

**Affiliations:** ^1^Department of Pediatric Surgery, Tianjin Medical University General Hospital, Tianjin, China; ^2^Faculty of Arts and Science, University of Toronto, Toronto, ON, Canada; ^3^The First Clinical Medical School, Shanxi Medical University, Taiyuan, China; ^4^Department of Colorectal Surgery, Tianjin Union Medical Center, Tianjin, China

**Keywords:** antioxidant, dietary complex antioxidant index, constipation, National Health and Nutrition Examination Survey, oxidative stress

In the published article, there was an error in the author list. The author, Daqing Sun, was placed as the second to last author, instead of the last author. The corrected author list appears below.

Wei Sun, Yuchao Wang, Lu Han, Yinshi Liu, Hongru Liu, Yunbing Tong, Ziying Jiang, Chen Xu, and Daqing Sun.

In the published article, there was an error regarding the affiliation for the author, Yinshi Liu. Instead of being affiliated with affiliation 2: Department of Colorectal Surgery, Tianjin Union Medical Center, Tianjin, China. Their correct affiliation should be 2: Faculty of Arts and Science, University of Toronto, Toronto, Canada.

In the published article, there was an error regarding the affiliation for the author, Hongru Liu. Instead of being affiliated with affiliation 1 and 2: Department of Pediatric Surgery, Tianjin Medical University General Hospital, Tianjin, China and Department of Colorectal Surgery, Tianjin Union Medical Center, Tianjin, China. Their correct affiliation should be 3: The First Clinical Medical School, Shanxi Medical University, Taiyuan, China”.

In the published article, there was an error regarding the affiliation for the author, Chen Xu. Instead of being affiliated with affiliation 2: Department of Colorectal Surgery, Tianjin Union Medical Center, Tianjin, China. Their correct affiliation should be 4: Department of Colorectal Surgery, Tianjin Union Medical Center, Tianjin, China

The authors apologize for this error and state that this does not change the scientific conclusions of the article in any way. The original article has been updated.

